# A Cross-Sectional Study of the Haematological Profile of Patients With Chronic Liver Disease (CLD)

**DOI:** 10.7759/cureus.40003

**Published:** 2023-06-05

**Authors:** Deepika Joshi, Mohamad Akram, Kunal Das, Mansi Kala

**Affiliations:** 1 Internal Medicine, Swami Rama Himalayan University, Dehradun, IND; 2 Pediatrics, Swami Rama Himalayan University, Dehradun, IND; 3 Pathology, Himalayan Institute of Medical Sciences, Swami Rama Himalayan University (SRHU), Dehradun, IND

**Keywords:** haemoglobin, child pugh score, thrombocytopenia, chronic liver disease, anaemia

## Abstract

The liver keeps haematological parameters normal and preserves haemostasis by storing iron, vitamin B-12, and, folic acid, necessary for healthy haematopoiesis. Anaemia of various aetiologies affects approximately 75% of chronic liver disease (CLD) patients, specifically caused by iron deficiency, hypersplenism, chronic diseases, autoimmune haemolysis, folic acid deficiency, aplasticity, and as a side effect of antiviral drugs. This study sought to observe the derangements in haematological parameters in patients with CLD, analyse the spectrum of anaemia in patients with CLD, and predict CLD outcomes utilizing Child-Pugh Score. This cross-sectional observational research was carried out in the Department of General Medicine, Himalayan Institute of Medical Sciences (HIMS), Dehradun, India over the course of a year. The patients with CLD who were admitted to the ward participated in the study. Most patient’s blood pictures reported normocytic normochromic with thrombocytopenia (TCP) (28.7%), macrocytic hypochromic with TCP (26%), microcytic hypochromic with TCP (13.3%) and macrocytic normochromic with TCP (9.3%). The incidence of anaemia was 85.3%: mild in 12.7% patients, moderate in 55.3% patients, and severe in 17.3% patients. Interestingly, this study also builds upon others suggesting that 85.9% of CLD patients have Class C Child-Pugh Score.

## Introduction

The liver is the most expansive and complex organ in terms of its functioning. In the human body, it performs a wide variety of activities including but not limited to the breakdown of carbohydrates, proteins, and lipids, neutralisation of a wide variety of toxins, metabolization of exogenous drugs and hormones, production of plasma proteins, and maintenance of the immune system, specifically the Kpuffer cells. In foetal life, liver is the major site of haematopoiesis, contrary to postnatal life, where it maintains haematological haemostasis. It further plays a significant role in the regulation of blood pressure and cascades secreting clotting factors and inhibitors, in addition to serving as a storage depot for iron, folic acid, and vitamin B-12 [1]. It plays a major role in keeping haematological parameters within normal and preserving haemostasis by storing iron, vitamin B-12, and folic acid-essential components of haem-iron metabolic pathways. Liver ailments typically manifest distinct clinical patterns and are typically categorised as hepatocellular diseases such as viral hepatitis, and alcoholic liver disease. However, in an event of a mixed pattern, signs of both hepatocellular and cholestatic damage occur concurrently - a typical case of a cholestatic variant of viral hepatitis observed in many drug-induced liver disorders [2]. Jaundice, tiredness, itching, pain in the right upper quadrant, nausea, abdominal distention, loss of appetite, and intestinal haemorrhage are common signs of liver disease [2].and 

Chronic liver disease (CLD) causes slow wear and tear of the liver echotexture leading to fibrosis and cirrhosis, both being conferred end stages of the illness. Concurrent complications such as bleeding and infection can lead to an increase in morbidity and death rates. Sometimes, it is connected with haematological abnormalities. CLD can have a myriad of underlying causes including but not limited to dietary deficiencies, bleeding, excessive use of alcohol, and abnormalities in the production of hepatic proteins or proteins involved in blood formation or coagulation. Overall CLD is linked to a broad spectrum of different haematological disorders [3]. More specifically, the blood picture gets affected by hepatocellular failure, portal hypertension, and jaundice. Hypersplenism is commonly associated with CLD. Erythrocyte survival is frequently reduced. In addition, issues with blood coagulation may be brought on by parenchymal liver illness as well as by cholestatic jaundice. The intricacy of the issue is compounded by the fact that dietary inadequacies, drunkenness, bleeding, and “problems associated with the hepatic synthesis of proteins needed in blood formation or coagulation” are all contributing factors [4]. Individuals who have liver illness, and a history of bleeding following mild trauma like venepuncture, as well as spontaneous bleeding, bruising, and purpura, are more relevant signs of a bleeding propensity than lab testing [4]. Fatigue is the key symptom of liver disease and the one that manifests most often. Manier times it is manifested as vague and perceived by the patients as drowsiness, weakness, listlessness, fatigue, need for sleep, a lack of endurance, and low energy levels. Notably, it is “afternoon” fatigue rather than “morning” tiredness - due to exhaustion - that often appears after activity or exercise and is sometimes evident or grave after proper rest; hence, referred to as “afternoon” fatigue. Fatigue is typically sporadic and fluctuates in intensity as time passes by in patients who suffer from liver disease [5]. The discomfort or aching in the right upper quadrant, sometimes known as “liver pain”, may be caused by a variety of liver illnesses. Typically, pain is accompanied by soreness across the liver region, specifically, the Glisson's capsule, which encircles the liver, and is densely packed with nerve endings. Stretching and irritation of Glisson's capsule is the primary source of the discomfort [5]. Another key haematological finding is the persistence of anaemia of various aetiologies which affect approximately 75% of CLD patients.

Anaemia in CLD is caused by iron deficiency, hypersplenism, chronic disease anaemia, autoimmune haemolytic anaemia, folic acid deficiency, aplastic anaemia, and as a side effect of antiviral drugs [6]. CLD compromises the synthetic functions of the liver, particularly hepcidin, a key protein in iron metabolism, whose overload leads to iron deposits in the liver [7]. Seventy-five percent of people with CLD get anaemia. In simple cirrhosis, it is typically of moderate severity & either normochromic normocytic, or mildly macrocytic. Microcytic hypochromic anaemia may develop if cirrhosis is exacerbated by bleeding or haemolysis [7]. In individuals who have liver illness, a history of bleeding following mild trauma like venepuncture, as well as spontaneous bleeding, bruising, and purpura, are more relevant signs of a bleeding propensity than lab testing [4]. In a nutshell, anaemia of CLD [8,9] can be accompanied by anaemia of chronic disease, iron loss (blood loss), folate deficiency, hypersplenism, aplastic anaemia (viral hepatitis is rare), sideroblastic (alcohol), disseminated intravascular coagulation (DIC) (rare), microangiopathy, and autoimmune origin (rare). As such, in view of the high prevalence of anaemia and thrombocytopenia (TCP) in CLD patients, the present study had three primary objectives: (1) To observe the derangements in haematological parameters in patients with CLD, (2) to analyse the spectrum of anaemia in patients with CLD, and (3) to predict CLD outcome utilizing Child-Pugh Score.

## Materials and methods

Participants

This cross‑sectional study was reported in accordance with the Strengthening the Reporting of Observational Studies in Epidemiology (STROBE) Statement, that is, the guidelines for reporting observational studies. It was carried out in the Department of General Medicine at the Himalayan Institute of Medical Sciences (HIMS), Swami Rama Himalayan University (SRHU) in Swami Ram Nagar, Dehradun, India. Ethics approval (Project no: SRHU/HIMS/RC/2023/110) was granted prior to recruitment, all participants provided written informed consent, and we adhered to our protocol to the Declaration of Helsinki. The patients with CLD, who were admitted to the HIMS in-patient ward participated in the observational study. Subjects were recruited for detailed history, thorough clinical examination, and investigations as per the working proforma. Patients more than 18 years age belonging to either gender with CLD due to: chronic hepatitis-C, chronic hepatitis-B, non-alcoholic steatohepatitis (NASH), primary biliary cirrhosis, autoimmune hepatitis, Wilson’s disease, and haemochromatosis were included in the study. However, patients diagnosed with acute hepatitis, drug-induced acute hepatic injury, fulminant hepatic failure, malignancy at the time of presentation, and jaundice associated with seasonal viral infection were excluded from the study sample.

Study design

Structured data collection form was used to record patient data as follows: Haematological parameters: haemoglobin, total leucocyte count (TLC), differential leucocyte count (DLC), packed cell volume (PCV), mean corpuscular volume (MCV), mean corpuscular haemoglobin concentration (MCHC), reticulocyte count, platelet count and mean platelet volume, Red cell distribution width (RDW). All cases of a CLD diagnosed in the Medicine out-patient door (OPD) and inpatient door (IPD) satisfying the inclusion criteria were taken. CLD was defined if the following parameters were fulfilled (patient was excluded if not stable or in shock, sepsis, or fulminant hepatic failure). Anaemia is categorized as (1) mild anaemia ranges <13 g/dL to 10 g/dL, (2) moderate between 7 g/dL to 10 g/dL, and (3) severe anaemia below 7 g/dL. Patients were diagnosed with TCP if their platelet count was less than 150 x 10^9^/L. The outcome of the patients was categorised as favourable (improved and discharged) or unfavourable (expired or leaving against medical advice due to non-improvement). The outcome groups so formed were compared with regard to haematological and biochemical parameters.

Statistical analysis

Excel for Microsoft Windows was used to enter the data, and the Statistical Package for Social Sciences (SPSS) was to do the statistical analysis. To investigate the distribution of a number of categorical and quantitative variables, a descriptive statistical analysis was carried out in the SPSS. To summarise categorical data (haematological parameters, Child-Pugh score), we used frequency (%) and mean ± standard deviation. One-way Analysis of variance (ANOVA) was used to evaluate whether there is a statistically significant difference between categories of anaemia (mild, mild, moderate, and severe). If the p-value is less than 0.05, the experiment is regarded to be statistically significant.

## Results

Participants demographics

Most patients belonged to the age group of 41-50 years (30%) and 51-60 years (28%). This was followed by 31-40 years (18%), 61-70 years (12%), 22-30 years (8.7%) and 71-80 years (3.3%). The mean age was 48.23 ± 12.15 years. Males and females were 88% and 12% respectively. From an occupation viewpoint, the majority of the patients were shopkeepers (27.3%), retired personnel (16%), and farmers (15.3%).

Haematological profile

Table 1 presents the distribution of CLD patients based on haematological profile. The majority of the patients showed normocytic normochromic with TCP (28.7%), macrocytic hypochromic with TCP (26%), microcytic hypochromic with TCP (13.3%) and macrocytic normochromic with TCP (9.3%). This specifically highlights the fact that anaemia blood picture largely depends upon the etiology and that exhibited a mixed pattern in most cases.

**Table 1:** Distribution of CLD patients based on haematological profile; macrocytic (larger RBC cell size), microcytic (smaller RBC cell size), normochromic (normal RBC colour), and hypochromic (light RBC colour) TCP: Thrombocytopenia

Haematological Parameters	Frequency (n)	Valid Percentage (%)
Macrocytic type with neutrophilic leucocytosis with TCP	1	0.7
Dimorphic picture (normocytic to macrocytic)	1	0.7
Dimorphic picture (normocytic to macrocytic), neutrophilic leucocytosis,	1	0.7
Hypochromic microcytic	1	0.7
Macrocytic hypochromic with TCP with spur cells	1	0.7
Macrocytic hypochromic	2	1.3
Macrocytic hypochromic with target cells	1	0.7
Macrocytic hypochromic with TCP	39	26.0
Macrocytic normochromic	1	0.7
Macrocytic normochromic with TCP	14	9.3
Microcytic hypochromic	1	0.7
Microcytic hypochromic with target cells	1	0.7
Microcytic hypochromic with TCP	20	13.3
Mild anaemia macrocytic type with neutrophilic leucocytosis with TCP	1	0.7
Moderate anaemia macrocytic type	1	0.7
Moderate anaemia	1	0.7
Moderate anaemia, macrocytic type	1	0.7
Moderate anaemia, normocytic normochromic, leukopenia, TCP	1	0.7
Normochromic normocytic	3	2.0
Normochromic normocytic with TCP	3	2.0
Normocytic hypochromic with TCP	5	3.3
Normocytic normochromic with TCP	43	28.7
Severe anaemia microcytic hypochromic type	2	1.3
Spherocytes with TCP	1	0.7
Spurr cells	1	0.7
Target cell	1	0.7
TCP	2	1.3

Table 2 presents association of Hb, TLC, HCT, MCV, MCHC, reticulocyte count, platelet count, prothrombin time, and RDW with the severity of anaemia. Even though there was significant difference in Hb concentration between different categories of anaemia (<0.001), following Bonferroni correction, there were no significant differences (Normal vs Mild vs Moderate vs Severe). Most blood parameters, including the red blood cells, white blood cells, or haemostasis parameters deter with anaemia severity, only Hb (g/dL) significantly plummets across anaemia severity.

**Table 2:** Association of Hb, TLC, HCT, MCV, MCHC, reticulocyte count, platelet count, prothrombin time, RDW with the severity of anaemia Hb: Haemoglobin, TLC: Total leucocyte count, HCT: Haematocrit, MCV: Mean corpuscular volume, MCHC: Mean corpuscular haemoglobin concentration, RDW: red cell distribution width.

Severity of Anaemia Mean ± SD	Normal	Mild	Moderate	Severe	p
Hb (g/dL)	12.02 ± 1.40	10.10 ±1.33	8.64 ± 1.00	5.56 ± 0.83	<0.001
TLC (per micrlitres)	9.36 ± 4.10	8.47 ± 2.98	10.10 ± 3.87	9.59 ± 3.96	0.39
HCT (per 100%)	26.10 ± 5.94	27.63 ± 5.51	26.18 ± 5.24	23.65 ± 4.86	0.09
MCV (femtolitre)	92.05 ± 9.73	89.67 ± 15.27	89.69 ± 11.05	86.66 ± 14.95	0.49
MCHC (g/dL)	28.78 ± 5.39	29.44 ± 4.23	29.02 ± 5.19	29.32 ± 4.24	0.97
Reticulocyte count (per microliter)	1.56 ± 0.51	1.78 ± 0.36	1.81 ± 0.36	1.82 ± 0.62	0.12
Platelet count (per microliter)	86.38 ± 7.16	70.78 ± 2.61	64.10 ± 8.50	63.67 ± 7.42	0.11
Prothrombin time (sec)	18.48 ± 4.25	15.53 ± 3.59	17.18 ± 4.50	18.80 ± 5.49	0.07
RDW (per 100%)	19.75 ± 2.27	19.26 ± 1.69	18.81 ± 2.35	19.27 ± 1.90	0.30

Table 3 presents a significantly higher Class C Child-Pugh Score: 69 (85.9%), the class-based predictor of severity of long-term liver disease, amongst the CLD patients. This highlights the fact that majority of CLD cases exhibit Class C (severe) Child-Pugh score, which suggests advanced hepatic dysfunction. Thus, a beforehand indication of severe liver damage will help clinicians to map out a treatment plan more effective in future.

**Table 3:** Distribution of CLD patients per the Child-Pugh Score

Child-Pugh Score	Frequency (n)	Valid Percentage (%)
Class A	5	3.4
Class B	26	10.7
Class C	69	85.9

**Figure 1:**
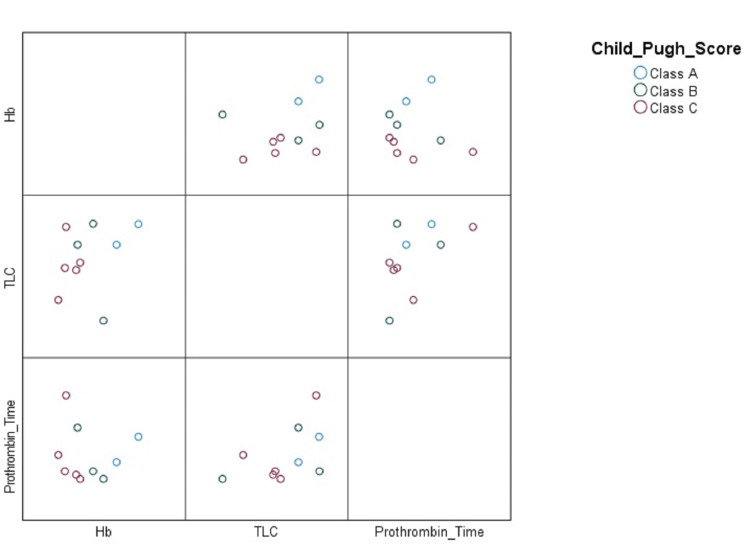
Correlation between haematological parameters and Child-Pugh Score Hb: haemoglobin, TLC: total leucoctye count, Prothrombin time

Figure 1 depicts correlation between haematological parameters of haemoglobin, TLC, and prothrombin time with Child-Pugh Score. A wide disarrayed picture is observed using the scatter-plot, with Class C score positively correlated with most haematological parameters.

## Discussion

This study has three primary objectives: to observe the derangements in haematological and biochemical parameters in patients with CLD, to analyse the spectrum of anaemia in patients with CLD, and to establish the association between the aetiology of CLD, and the type and severity of anaemia. Our results showed a haematological profile of CLD patients largely depends upon the aetiology and that has exhibited a mixed pattern in most cases. Most blood parameters, including the red blood cells, white blood cells, or haemostasis parameters although deter, only Hb (g/dL) significantly plummets across anaemia severity. And Child-Pugh score suggests advanced hepatic dysfunction across most CLD cases.

Pathophysiology: haematological derangement in CLD

Patients who suffer from alcoholic liver cirrhosis frequently present with abnormalities in their haematological parameters. There are multiple causes that contribute to the development of aberrant haematological indices. These variables include portal hypertension-induced sequestration, changes in bone marrow stimulating factors, and virus and toxin-induced bone marrow suppression. Consuming excessive amounts of alcohol produces direct suppression of the bone marrow, which in turn leads to harmful effects on the blood cell lines. Indirectly, it interferes with the patient's nutritional biology, which ultimately leads to the generation of cells that are not fully mature in their functions. Alcohol consumption creates complex aberrations in people with Haematological indices, which can lead to lethal consequences and an increased risk of death for these patients [6]. Abnormalities in the Haematological indices are connected with a greater risk of consequences, including bleeding and infection [8].

Comparison to literature: haematological derangements in CLD

In our study, the majority of the patient’s blood picture showed normocytic normochromic with TCP (28.7%), macrocytic hypochromic with TCP (26%), microcytic hypochromic with TCP (13.3%) and macrocytic normochromic with TCP (9.3%). In our study, the majority of patients have the normocytic normochromic type of anaemia which is the most common anemia found in alcoholics followed by macrocytic hypochromic anaemia. It was found that TCP is almost always present in all patients of CLD, irrespective of the etiology. Similar results were seen in a study conducted by Tara et al. [9], the most prevalent type of anemia that was observed was normocytic normochromic anaemia (25.8%). In our study, anaemia was mild in 2.7% patients, moderate in 55.3% patients, and severe in 17.3% patients. According to Khan et al. [10], 42.6% of persons had mild anaemia (Hb in the range of 9.5-13 g/dL), whereas 26.1% of patients had severe anaemia (8 g/dL), and a comparable percentage as in our study had moderate anaemia (8-9.5 g/dL). Anaemia was found to be present in 109 out of 115 individuals, which is a prevalence rate of 94.8%, according to a study that was carried out in 2015 and 2016 by Khan et al. [10]. According to the findings of a study carried out by Rosario et al., 75% of CLD patients were anaemic [11]. In our study, an association of haemoglobin with the severity of anaemia was found to be statistically significant. In our study, the incidence of anaemia was 85.3%. It was found that patients with normal range (14.7%), mild anaemia were 12.7%, moderate (55.3%) and severe (17.3%). According to the findings of research that was carried out by Kaur et al. [12], the mean Hb level was 8.8 g/dL. There was a total of 90 patients, 12 of whom had Hb levels that were within the normal range (13.3%), 10 of whom had mild anaemia (11.1%), 29 of whom had moderate anaemia (32.2%), and 39 of whom had severe anaemia (43.3%). According to a study conducted by Sambyal et al. [13], 13.9% of patients had a haemoglobin level that was lower than 7g%.

In our study, an association of TLC with the severity of anaemia was found to be statistically not significant. Patients in the normal range have TLC (9.36 ± 2.98), mild anaemia with TLC (8.47 ± 2.98), moderate anaemia with TLC (10.10 ± 3.87) and severe anaemia with TLC (9.59 ± 3.96). According to the findings of research that was carried out by Sambyal et al. [13], the total WBC count in 42 patients was lower than 4,000 cells/mm^3^, while the counts of 89% of patients were normal (4,000- 11,000 cells/mm^3^). Leucocytosis was found to be present in 3.3% of the individuals who were examined. There was no link found between the TLCs and either the presence of splenomegaly or the amount of time it took for the patient to bleed. Neither of these factors affected the length of time it took for the patient to bleed. Because leukopenia and leucocytosis are more prevalent in persons who have infections, such as urinary tract infections and spontaneous bacterial peritonitis, it is essential to understand the reasons behind why this is the case. 20.9% of patients displayed leucocytosis (TLC>11 x 10^9^), whereas 17.4% of patients demonstrated leukopenia, as stated by Khan et al. [10] (TLC4 x 10^9^). According to Kaur et al. [12], the mean TLC (in mm^3^) and the mean platelet count (x10^3^/micro-L) were, respectively, 11,445 and 151. Both of these numbers are expressed in millimetres per cubic inch. Out of a total of 90 individuals, leucocytosis was seen in 41 patients (45.6%), whilst leukopenia was identified in 14 patients (15.6%). In our study, an association of MCV with the severity of anaemia was found to be statistically not significant. In the study done by Kaur et al. [12], microcytic anaemia was present in 21.1% of the patients. The highest possible number of patients, 58 (64.4%), was within the normocytic range. The average MCV was 84.95 fL.

In our study, an association of platelet count with the severity of anaemia was found to be statistically not significant. We found that the patient having normocytic anaemia was also found to have more TCP. So, the percentage and the severity of TCP are present in the normocytic type of anaemia in patients of CLD, irrespective of the ethology. Maximum patients of CLD were found to have TCP, irrespective of ethology and no association was found with the severity of anaemia. In our study, 60 % of the patients have TCP and in a similar study conducted by Kaur et al. [12] TCP was seen in 58.9% of patients. In the same way, the platelet count was normal in the early phases of the research that was carried out by Sambyal et al. [13], but a declining trend of platelet count was found as the severity of CLD increased. TCP was seen in 48.7% of the patient population. CLD frequently manifests with abnormalities in the coagulation system. In our study, prothrombin time was in the range of 17.44 ± 4.62 and INR was in the range of 2.54 ± 0.90. In the study that was done by Kaur et al. [12], 88.9% of the patients exhibited a prolonged prothrombin time international ratio (PT-INR), which is indicative of a lack of clotting factor. 72% of patients had a prothrombin time that was extended by more than six seconds. The overall average international ratio (INR) was 1.92 [12]. Jha et al. [14] and Raja et al. [15] found haematological derangements, specifically leukocytosis, in CLD patients more prevalent than leucopenia and TCP; with the total number of white blood cells ranging anywhere from 1050/mm3 to 16,100/mm3 [15]. Jha et al. also observed increased prothrombin time and activated partial thromboplastin time (APTT) [14]. However, contrary to expected, Acharya et al. [16] 33.33% cirrhotic patients exhibited leucopenia, which may be brought on by hypersplenism. Further, in an investigation by Das et al. [17], platelet count was much lower in alcoholics, whereas PT/INR was significantly greater in alcoholic liver disease patients. And the median number of leucocytes in the population was 7,500 cells per mm^3^ according to Jain et al. [18].

As a whole, it is important to underscore that the results of this study should not be interpreted as to demonstrate a clear pattern of specific haematological derangements. Indeed, there is still a need to evaluate the haematological profile, specifically in the context of alcohol-related liver disease (ALD) and non-alcoholic fatty liver disease (NAFLD); however, it will take time until that happens.

Clinical implications and management

Whilst evaluating CLD, non-invasive assessment of fibrosis and cirrhosis is highly demanded. Further, identification of haematological derangements in NAFLD, conditions caused by a build-up of fat in the liver, specifically NASH is needed. Additionally, the search for novel medications to improve the prognosis of NAFLD is urgently warranted.

Limitations and future directions

The cause of anaemia in individual patients has not been extensively studied which could make a difference in the treatment approach. This specifically pertains to iron, vitamin B12, and folic acid deficiencies. A more type-specific analysis would underpin the treatment plan per se. Also, multivariate analysis of the co-variates could add a new perspective to anaemia in CLD. Since data collection was explicitly based on hospital questionnaires, the paucity of some key parameters constrained us from performing further multivariate analysis. Under-representation of women (only 12%) in our sample can be explained by the fact that CLD prevalence is much larger in men than women, perhaps due to higher alcohol consumption. Further studies with a greater number of patients, particularly of different ethnicities from across the globe will surely bolster the results of this study.

## Conclusions

The complete blood count had shown that the majority of patients had anaemia, leukopenia, and TCP. The incidence of anaemia was 85.3%: mild in 12.7% patients, moderate in 55.3% patients, and severe in 17.3% patients. Interestingly, this study also builds upon others suggesting that 85.9% of CLD patients have Class C Child-Pugh Score.
